# Supplementary figures and images for: Individuals with latent tuberculosis in a high TB endemic country show mild COVID-19
Source: PLoS One. 2025 Dec 30;20(12):e0339240. doi: 10.1371/journal.pone.0339240 (PMC12753056; doi:10.1371/journal.pone.0339240)

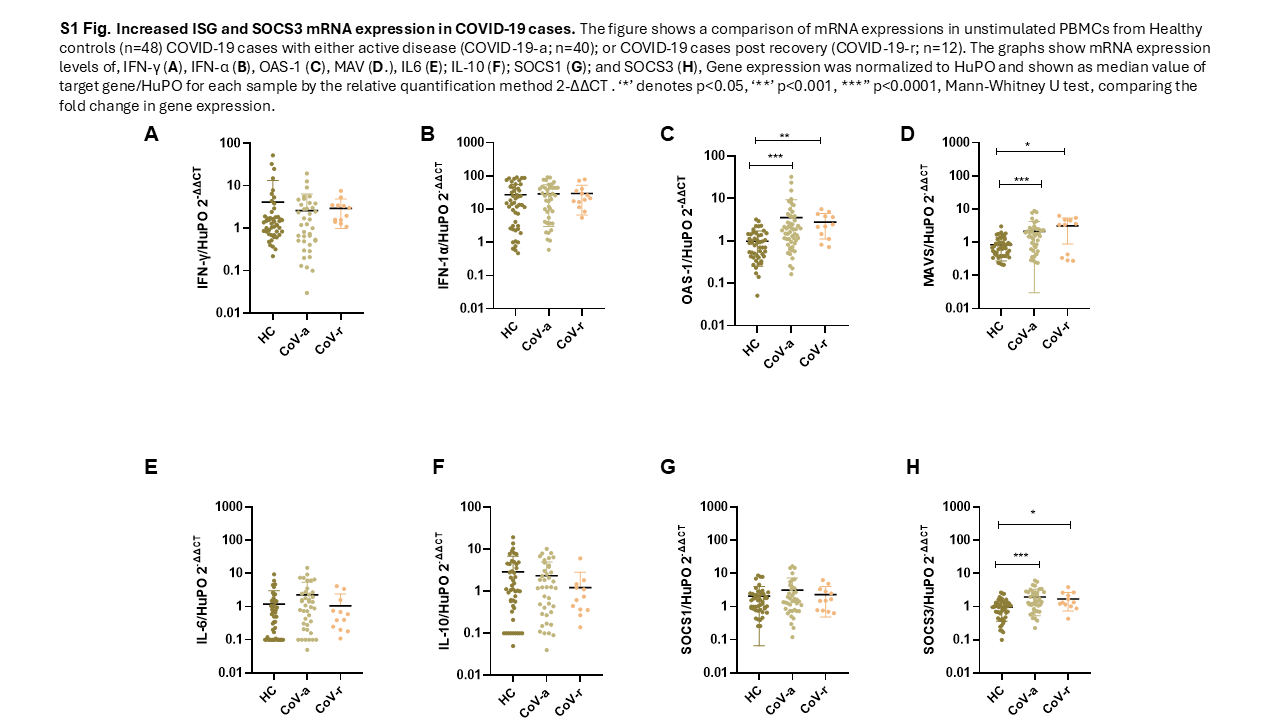

Supplement: S1 Fig — (TIF) [file pone.0339240.s001.tif]

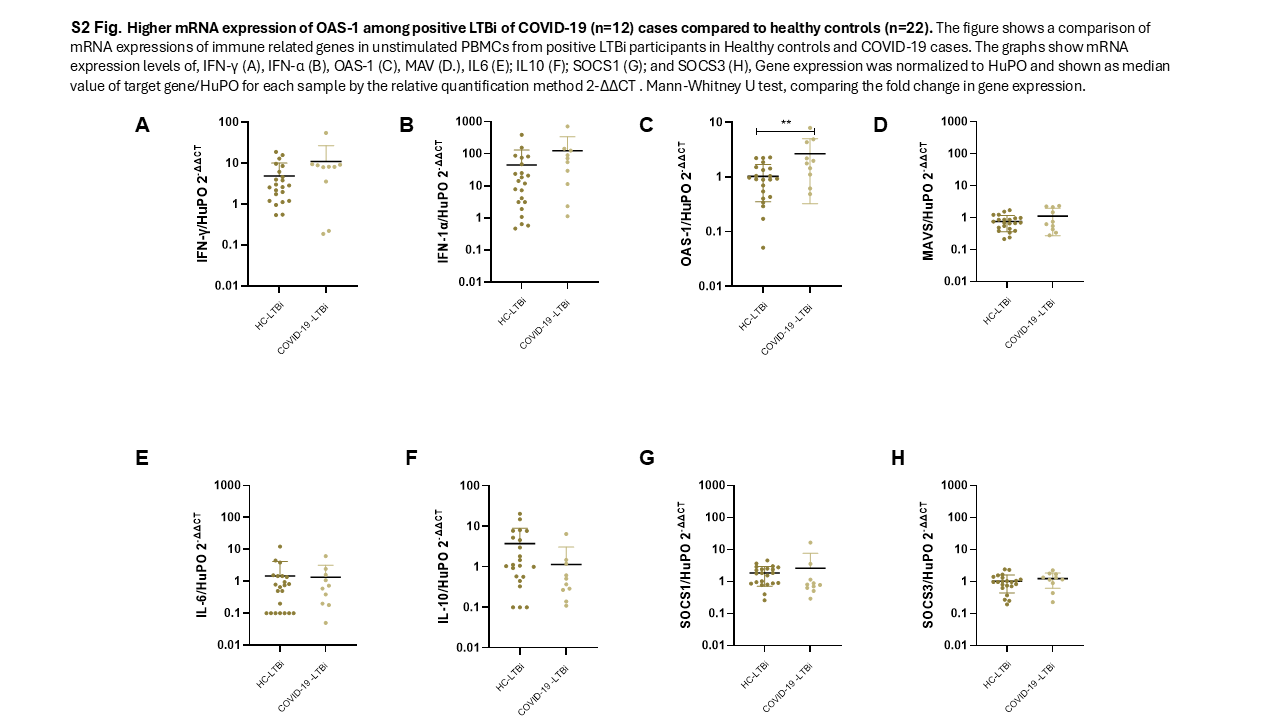

Supplement: S2 Fig — (TIF) [file pone.0339240.s002.tif]
