# Supplementary material for: Individuals with latent tuberculosis in a high TB endemic country show mild COVID-19
Source: PLoS One. 2025 Dec 30;20(12):e0339240. doi: 10.1371/journal.pone.0339240 (PMC12753056; doi:10.1371/journal.pone.0339240)
Supplement: S1 Table — (PDF) [file pone.0339240.s003.pdf]

**S1 Table. Details of primers used for RT-PCR testing**

| S. No | Seq Name | | Seq 5' to 3" | Melting Temperature (TM) in $^{O}C$ |
|---|---|---|---|---|
| 1 | HuPO | FWD | GCTTCCTGGAGGGTGTCC | 65 |
| | | REV | GGACTCGTTTGTACCCGTTG | |
| | IFN-γ | FWD | ATTCGGTAACTGACTTGAATGGCG | 60 |
| | | REV | CTCTTCGACCTCGAAACAGC | |
| 3 | IFN-1α | FWD | CTTGGATTCCTACAAAGAAGCAGC3′ | 64 |
| | | REV | TCCTCCTTCTGGAACTGCTGCA3′ | |
| 4 | OAS-1 | FWD | AGGAAAGGTGCTTCCGAGGTAG3′ | 64 |
| | | REV | GGACTGAGGAAGACAACCAGGT3′ | |
| 5 | MAVS | FWD | ATGGTGCTCACCAAGGTGTCTG3′ | 64 |
| | | REV | TCTCAGAGCTGCTGTCTAGCCA3′ | |
| 6 | IL-6 | REV | CATCCATCTTTTTCAGCCAT | 60 |
| | | FWD | ATGTAGCCGGCCCACACAGA | |
| 7 | IL-10 | FWD | TGAGAACCAAGACCCAGACA | 62 |
| | | REV | TCATGGCTTTGTAGATGCCT | |
| 8 | SOCS1 | FWD | TTTTTCGCCCTTAGCGTGA-3′ | 62 |
| | | REV | AGCAGCTCGAAGAGGCAGTC-3′ | |
| 9 | SOCS3 | FWD | GGCCACTCTTCAGCATCTC | 62 |
| | | REV | ATCGTACTGGTCCAGGAACTC | |
