# Supplementary material for: Individuals with latent tuberculosis in a high TB endemic country show mild COVID-19
Source: PLoS One. 2025 Dec 30;20(12):e0339240. doi: 10.1371/journal.pone.0339240 (PMC12753056; doi:10.1371/journal.pone.0339240)
Supplement: S2 Table — (PDF) [file pone.0339240.s004.pdf]

S2 Table. Comparison of characteristics of positive LTBi individuals in Healthy Control and COVID-19 groups.

		Healthy controls -LTBi positive (n=47)	COVID-19-LTBi positive (n=23)	*p* value
**Age (years)**	≤ 50 years	25 (53.2%)	19 (82.6%)	0.409
	> 50 years	22 (46.8%)	4 (17.4%)	
**Gender**	Female	22 (46.8%)	12 (52.1%)	0.758
	Male	25 (53.2%)	11 (41.9%)	
**BCG scar**	Present	35 (74.4%)	16 (69.5%)	0.973
**History of TB Contact**	Yes	7 (14.8%)	1 (4.3%)	0.642
**Comorbidity**	Diabetes	3 (6.3%)	1 (4.3%)	0.844
	Others	8 (17%)	3 (13%)	

BCG, bacille Calmette-Guerin vaccination. **Other comorbidities included hypertension, thyroid disorders, cancers, cardiac diseases and asthma. Statistical analysis between groups was performed using either Mann-Witney test, or the chi square test. *Differences are significant at $p < 0.05$ at 95% CI.
