# Supplementary material for: Individuals with latent tuberculosis in a high TB endemic country show mild COVID-19
Source: PLoS One. 2025 Dec 30;20(12):e0339240. doi: 10.1371/journal.pone.0339240 (PMC12753056; doi:10.1371/journal.pone.0339240)
Supplement: S3 Table — (PDF) [file pone.0339240.s005.pdf]

S3 Table. mRNA expression in HC and COVID-19 study groups with and without LTBi

**Gene target**	**Healthy controls (n=48)**		
	LTBi +ve (n=22)	LTBi -ve (n=26)	***p value***
IFN-γ	2.728 (4.61)	2.364 (1.51)	0.861
IFN-1α	13.12 (6.71)	15.39 (9.12)	0.718
OAS-1	0.919 (6.31)	0.635 (4.15)	0.476
MAVS	0.695 (2.31)	0.480 (1.87)	0.801
IL-6	0.705 (0.98)	0.34 (0.63)	0.913
IL-10	1.10 (4.7)	1.06 (2.91)	0.461
SOCS1	1.812 (7.26)	1.305 (5.17)	0.597
SOCS3	0.96 (1.19)	1.036 (2.21)	0.739
	**COVID-19 cases (n=40)**		
**Gene target**	LTBi +ve (n=12)	LTBi -ve (n=40)	***p value***
IFN-γ	8.14 (3.19)	3.27 (2.81)	0.352
IFN-1α	25.89 (3.12)	13.13 (7.13)	0.254
OAS-1	1.87 (5.67)	1.55 (3.13)	0.74
MAVS	1.056 (1.2)	0.680 (14.81)	0.526
IL-6	0.66 (0.96)	0.749 (0.75)	0.762
IL-10	0.44 (0.98)	1.24 (1.28)	0.108
SOCS1	0.79 (1.16)	1.446 (2.34)	0.233
SOCS3	1.23 (0.51)	1.29 (0.81)	0.366

The table shows the median (IQR) of mRNA expression in PBMCs of HC (healthy controls) and COVID-19 groups calculated by the relative quantification method 2-ΔΔCT in study participants. The Mann-Whitney U test was applied to check the difference in expression level. P<0.05 was considered as significant.
