# Supplementary material for: Individuals with latent tuberculosis in a high TB endemic country show mild COVID-19
Source: PLoS One. 2025 Dec 30;20(12):e0339240. doi: 10.1371/journal.pone.0339240 (PMC12753056; doi:10.1371/journal.pone.0339240)
Supplement: Attachment — Submitted filename: Point_by_Point_Response-zh.pdf [file pone.0339240.s007.pdf]

To

Academic Editor

PLOS ONE

Dear Dr. Yamaguchi,

This is to submit the revised manuscript titled **"Individuals with latent tuberculosis in a high TB endemic country show milder COVID-19 morbidity"** for consideration.

We are thankful to the editor and reviewers for taking out their time and efforts to bring important suggestions for our manuscript. We have tried our best to address the comments and made changes to the manuscript accordingly. These are identified in the table below.

Response to Editor:

S. No.	Comment	Response	Location
1.	Please ensure that your manuscript meets PLOS ONE's style requirements, including those for file naming.	We have made the required changes in document as per PLOS ONE's requirement.	
2.	During our evaluation of the documents provided, we noted that your ethics approval letter did not cover the entire date range for participant recruitment and data collection. Before we can proceed further with the submission, please provide the ethics approval extension document(s) for the study.	Thank you for highlighting this point. The samples recruited for this study were from two different projects where COVID-19 cases and Healthy controls were recruited. The combined study period of the work was from 4th January 2022 to 13th August 2023. The Ethics approval letters of both studies have been uploaded and the Ethics approval numbers allocated by the Ethics Review Committee, Aga Khan University have been mentioned in Methods.	
3.	Please provide an amended statement that declares *all* the funding or sources of support (whether external or internal to your organization) received during this study, as detailed online in our guide for authors	The amended statement of the funding has been provided in the cover letter.	Please refer to cover letter

Reviewer 1:

S. No.	Comment	Response	Location
4.	As this is a cross-sectional observational study, please avoid phrases like “LTBi confers protection” or “LTBi reduces severity.” Use terms such as “associated with” or “suggests a possible protective effect.”	Thank you for highlighting this point. The mentioned phrases have been removed from the manuscript and replaced with suggested words.	Edits on page 2, in abstract and conclusions.
5.	Include logistic regression to control for confounders such as age, sex, comorbidities, vaccination status, and time since infection. This is essential for substantiating your key conclusion.	Thank you for the suggestion. We have applied logistic regression between healthy controls and COVID-19 cases. This showed 54% protection from COVID-19 for LTBi positive individuals.<br>For subsequent data analysis (table 2), we could not use logistic regression as there were zero cases of LTBi in the hospitalized COVID-19 group. Therefore, we have applied Chi-squared analysis to investigate the association of specific variables.	Tables 1 and 2
6.	While the X.DOT-TB assay is explained, consider clarifying its comparability to standard QFT in the main Methods (not just Supplementary). It would also help to justify using only this assay for LTBi classification.	Thank you for this suggestion. We have now mentioned the comparison of X.DOT-TB with QFT in the main Methods. Also clarified that it this assay was used only for LTBi classification	Page 5-6, Methods
7.	BCG scar status was not associated with COVID-19 outcomes, but the Discussion still overemphasizes the “trained immunity” angle from BCG. Please reconcile this more clearly with your data.	Thank you. We have reduced the emphasis on BCG related trained immunity in the Discussion.	Page 14-16, Section Discussion
8.	The significance of raised OAS1 and SOCS3 in LTBi-positive COVID-19 cases is underexplained. Are these markers known to mediate antiviral responses in the TB context? Please elaborate or temper the discussion if speculative.	We have expanded the functions of OAS1, MAVS and SOC3 in the perspective of LTBi and COVID-19.	Page 15, 16, 18<br>Section Discussion

Reviewer 2:

S. No.	Reviewer Comment	Author Response	Reference
1.	I agree that the data presented appears to show that the severity of COVID-19 disease is reduced in people with LTBi but I do not think the data presented convincingly shows protection against SARS-CoV-2 infection. Notably in figure 3 E compares IgG levels in a small number of HC who were either LTBi- (n=30) or LTBi+ (n=30), a significant proportion of the LTB+ve patients are positive for anti-RBD antibodies. The difference between the groups in figure 3E does not look significant to me and if I understand correctly both groups appear to have been approximately equally likely to have been infected with SARS-CoV-2 (and presumably had mild symptoms). If I am correct, I would ask the authors to limit their claim of protection to protection from serious disease not protection from infection throughout the paper (introduction, discussion and conclusions).	Thank you for your comments. The IgG data shown in Figure 3 (now figure 4) is of the all HC (n=60) and COVID-19 (n=120) group participants measured using the RBD ELISA at 1/100 dilution as per our standard protocol.<br><br>The smaller group analysis (Fig 3D-F) is of IgG RBD titrations of 30 individuals each from the HC and COVID-19 groups included in 4A. This has been further explained in the text.	Page 12, section: Results
2.	Notably also in the conclusions section of the abstract “We suggest that the immune reactivity to Mtb is associated with an increased protection against SARS-CoV-2 as indicated by both the lower frequency of COVID-19 and the reduced severity of disease in LTBi positive individuals.” delete “lower frequency of COVID-19” from this statement.	We have rephrased the title and conclusions accordingly.	Page 1 title, Page 2, abstract, Page 17, conclusion.,
3.	Minor point CODID should be COVID in the title “Higher numbers of M. tuberculosis-specific T cells in HC than in CODID 19 LTBi positive individuals.”	We have corrected this typo.	Page 11, section results

*****
