# Supplementary material for: Individuals with latent tuberculosis in a high TB endemic country show mild COVID-19
Source: PLoS One. 2025 Dec 30;20(12):e0339240. doi: 10.1371/journal.pone.0339240 (PMC12753056; doi:10.1371/journal.pone.0339240)
Supplement: Attachment — Submitted filename: Reviewers PLOS one_R2.pdf [file pone.0339240.s008.pdf]

Dear Editor,

Thank you for giving us the opportunity to revise our manuscript. Please find below responses to comments made by the Reviewers.

Best wishes

Zahra Hasan

Response to Reviewers Comments

Reviewer #2: The authors have revised their paper and moderated the claims with respect to protection of infection and the potential protective effect of BCG. All the points raised have been addressed.

Ans. We thank reviewer 2 for their feedback and appreciation

Reviewer #3: This study compares the Mycobacterium tuberculosis–specific cellular immune response in patients with COVID-19 versus healthy individuals. The authors conclude that this immune response is significantly more pronounced in healthy individuals compared with COVID-19 patients.
Major Comments
1. 1. The lack of interferon production by T cells indicates a decrease in cellular immunity, which may also be attributable to the higher prevalence of chronic comorbidities in the patient group compared with healthy controls. This should be clearly reflected in the discussion.

Response:

Thank you for bringing up this point. We have referred to this point in the Discussion (l295-309).

2. 2. The selection of patients and controls may be subject to biases, which are not sufficiently explained in the Materials and Methods section. For example, the timing of COVID-19 patient recruitment varies considerably after infection, which could substantially influence cellular immune responses.
Response:

We have further explained the selection of patients and controls in the methods. The limitation of sample sizes of each cohort is discussed and as how the heterogeneity of the cohorts may contribute to the differences observed (l361-l376).

3. 3. The fact that some patients were hospitalized while others were managed on an outpatient basis likely contributed to heterogeneous immune responses. This should be acknowledged and discussed as a limitation of the study.

Response:

As mentioned above we have discussed this in the Limitations.

4. 4. In the Methodology section, the process of recruiting both patients and controls requires a more straightforward explanation.

Ans We have written up the process of recruitment of participants in more detail with inclusion and exclusion criteria for COVID-19 and Control groups.

Minor Comments

1. In the Abstract, the three introductory sentences are not entirely coherent with the primary study objective.

Response:

The abstract has been corrected.

2. The following sentence in the ~~Abstract~~ introduction is unclear: "Therefore, MTB infection in active TB patients, or individuals with latent MTB infection, are likely to experience immune modulation that may alter the consequence of SARS-CoV-2 infection?"

Response:

The abstract has been corrected.

3. The authors should clarify what the messenger RNA sequences for OAS1, MAVS, SOCS1, and SOCS3 represent.

Response: The sequences of primers used for RT-PCR to detect OAS-1, MAVS, SOCS1 and SOCS3 are given in S Table 1.

4. The description of the mRNA sequences analyzed should be expanded, and the primers used for real-time PCR should be specified.

Response:

The mRNA sequences were obtained from Eurofins (Fleuri, Luxembourg) and sequences were obtained from available literature and have been tested in our previous studies. The references and further details have been now mentioned in the methods.

5. For the method 'X. In the DOT-TB assay for screening LTBi, the antigens employed should be described.

Response:

The X DOT TB assay has ESAT-6 and CFP-10 antigens derived from MTB. This has been described in Method section.

6. In the phrase 'As per manufacturer's instructions', the manufacturer of the kit must be explicitly named.

Response:

TB Healthcare Biotechnology, China is the manufacturer of the kit. This has been mentioned now in the methods.

7. In the Results section, subheadings currently present the outcomes rather than the study objectives.

Response:

We have corrected subheadings in the Results section.

8. The entire manuscript requires a comprehensive revision of the English language, including correction of grammatical errors.

Response:

Thank you for your feedback. We have thoroughly revised the manuscript for English language and grammatical mistakes.

Reviewer #4: Overall, this is a well written revised manuscript with a few minor suggestions for revision;

INTRODUCTION:
1. Rephrase 'with 677 million cases and 6.9 million deaths reported worldwide to 10 March 2023 ---' as 'with 677 million cases and 6.9 million deaths reported worldwide by 10 March 2023 ---'

Response:

Thank you for highlighting the error. We have corrected the sentence as per the suggestion.

2. Define 'TST' at first use of term

Response:

We have defined Tuberculin Skin Test (TST) in the text.

3. Rephrase 'Currently, studies on the association between SARSCoV-2 and MTB are both scare' as 'Currently, studies on the association between SARSCoV-2 and MTB are both scarce'

Response:

We have replaced the word “scare with scarce”

METHODS:
4. You state that 'All 128 individuals in the COVID-19 group have been confirmed through a positive SARS-CoV-2 PCR test'. Further, was genotyping performed to ascertain the SARS-CoV-2 strain that was associated with COVID-19 at that time?

Response:

The SARS-CoV-2 strains from the participants in this cohort were not sequenced. However, at the time our group was involved in genomic surveillance, and we had reported that Omicron variants were predominant at the time of the study (1).

5. Further, for SARS-CoV-2 PCRs, did you use in-house or commercial kits?

Response:

No, the PCR was performed by the clinical laboratory of Aga Khan University Hospital and the cobas SARS-CoV-2 PCR assay was used, now detailed in Methods.

6. For RT-PCR on PBMCs, did you mean you did 'reverse-transcriptase real-time PCR' or just 'reverse-transcriptase PCR'? Please clarify.

Response:

Thank you we have clarified that we used reverse-transcriptase PCR using real time analysis for the study of mRNA expression of target genes. This has been clarified in Methods.

DISCUSSION:
8. In the limitations section, or somewhere in the discussion, comment on the fact that the phrase 'TB infection' is currently evolving from being understood as a dichotomy to a continuum, meaning the LTBi individuals could actually have been having TB disease? Just a thought.

Response:

Thank you for this point. We have added it to the Discussion and Conclusion.

7. Are the primer sequences in-house, i.e., designed by you, or did you use primer sequences published in literature?

Response:

The Methods section has additional information regarding the sources for the design of primers. Primers were procured from Eurofins (Fleuri, Luxembourg).

References

1. Bukhari AR, Ashraf J, Kanji A, Rahman YA, Trovao NS, Thielen PM, et al. Sequential viral introductions and spread of BA.1 across Pakistan provinces during the Omicron wave. BMC Genomics. 2023;24(1):432.
